# Repurposing of α1-Adrenoceptor Antagonists: Impact in Renal Cancer

**DOI:** 10.3390/cancers12092442

**Published:** 2020-08-28

**Authors:** Meredith Mihalopoulos, Zachary Dovey, Maddison Archer, Talia G. Korn, Kennedy E. Okhawere, William Nkemdirim, Hassan Funchess, Ami Rambhia, Nihal Mohamed, Steven A. Kaplan, Reza Mehrazin, Dara Lundon, Che-Kai Tsao, Ketan K. Badani, Natasha Kyprianou

**Affiliations:** 1 Department of Urology, Tisch Cancer Institute, Icahn School of Medicine at Mount Sinai, New York, NY 10029, USA; meredith.mihalopoulos@icahn.mssm.edu (M.M.); zachary.dovey@mountsinai.org (Z.D.); Maddison.Archer@mountsinai.org (M.A.); Talia.korn@mountsinai.org (T.G.K.); Kennedy.Okhawere@mountsinai.org (K.E.O.); wn442@nyu.edu (W.N.); hf1031@nyu.edu (H.F.); ar6326@nyu.edu (A.R.); nihal.mohamed@mountsinai.org (N.M.); steven.kaplan@mountsinai.org (S.A.K.); reza.mehrazin@mountsinai.org (R.M.); Dara.Lundon@mountsinai.org (D.L.); ketan.badani@mountsinai.org (K.K.B.); 2 Department of Oncological Sciences, Icahn School of Medicine at Mount Sinai, New York, NY 10029, USA; 3 Department of Medicine/Division of Hematology-Oncology, Icahn School of Medicine at Mount Sinai, New York, NY 10029, USA; che-kai.tsao@mssm.edu; 4 Department of Pathology and Laboratory Medicine, Icahn School of Medicine at Mount Sinai, New York, NY 10029, USA

**Keywords:** renal tumors, prevention, α1-adrenoceptor antagonists, anoikis, vascularity

## Abstract

Renal cancer ranks twelfth in incidence among cancers worldwide. Despite improving outcomes due to better therapeutic options and strategies, prognosis for those with metastatic disease remains poor. Current systemic therapeutic approaches include inhibiting pathways of angiogenesis, immune checkpoint blockade, and mTOR inhibition, but inevitably resistance develops for those with metastatic disease, and novel treatment strategies are urgently needed. Emerging molecular and epidemiological evidence suggests that quinazoline-based α1-adrenoceptor-antagonists may have both chemopreventive and direct therapeutic actions in the treatment of urological cancers, including renal cancer. In human renal cancer cell models, quinazoline-based α1-adrenoceptor antagonists were shown to significantly reduce the invasion and metastatic potential of renal tumors by targeting focal adhesion survival signaling to induce anoikis. Mechanistically these drugs overcome anoikis resistance in tumor cells by targeting cell survival regulators AKT and FAK, disrupting integrin adhesion (α5β1 and α2β1) and engaging extracellular matrix (ECM)-associated tumor suppressors. In this review, we discuss the current evidence for the use of quinazoline-based α1-adrenoceptor antagonists as novel therapies for renal cell carcinoma (RCC) and highlight their potential therapeutic action through overcoming anoikis resistance of tumor epithelial and endothelial cells in metastatic RCC. These findings provide a platform for future studies that will retrospectively and prospectively test repurposing of quinazoline-based α1-adrenoceptor-antagonists for the treatment of advanced RCC and the prevention of metastasis in neoadjuvant, adjuvant, salvage and metastatic settings.

## 1. Introduction: The Therapeutic Challenge

Renal cancer ranks twelfth in incidence among cancers worldwide and has a lifetime risk of 1 to 63 for a given individual, with numbers estimated to be increasing at a rate of 2.4% per year [1,2]. Globally, 6 in 100,000 males and 3 in 100,000 females are diagnosed annually with renal cancer, with the incidence estimated to be increasing at a rate of 2.4% per year [1,2] with limited therapeutic management options [3,4,5]. In the United States alone, over 60,000 individuals were diagnosed with renal cell carcinoma (RCC) in 2018, with an estimated 14,970 deaths resulting from the illness [6]. Despite the introduction of systemic targeted therapies, five-year survival rates for locally advanced and metastatic disease remain at 70% and 12%, respectively in the year 2020 [7].

The most common genetic abnormality for clear cell RCC (ccRCC) is the chromosome 3p deletion and inactivation of the von Hippel Lindau (VHL) tumor suppressor gene, present in almost all familial and up to 60% of sporadic RCCs [8,9]. Loss of the VHL gene leads to the upregulation of hypoxia-inducible factor (HIF) and activation of vascular endothelial growth factor receptors (VEGFR) and other signaling pathways, leading to tumorigenesis with an aggressive angiogenic phenotype [8,9]. Current treatment of RCC is based on the tumor stage at diagnosis. Localized disease, with or without evidence of regional spread, is typically managed surgically alone. For those with more advanced localized or loco-regional disease after nephrectomy, treatment with adjuvant VEGFR TKI sunitinib has been approved by the Federal Drug Administration, although the modest clinical benefit and concern for the potential side effects has largely limited its clinical application to data [10].

For metastatic ccRCC, a number of approaches combining immune checkpoint inhibitors or with VEGFR-TKIs have now become the standard of care after demonstrating a definitive survival benefit in the first-line setting [11], compared to sunitinib alone [2]. However, the optimal first-line and sequence of subsequent therapies are not well defined. The current systemic therapeutic approaches include: targeting pathways of angiogenesis, immune checkpoint blockade, and mTOR inhibition. Inevitably, treatment resistance is either intrinsic or eventually develops. New mechanistic approaches are urgently needed to improve survival outcomes in this patient population.

Investigative efforts from our group and others have focused on the role of quinazoline-derived α1-adrenoreceptor antagonists in the treatment of renal cancer. By inducing smooth muscle relaxation and vasodilation, these drugs are currently used in the treatment of hypertension (HTN) and renal and ureteric stones [12,13], as well as of benign prostatic hyperplasia (BPH) [13]. More recently the potential efficacy of these drugs in the treatment of prostate cancer has been proposed, due to the ability to induce apoptosis and overcome anoikis resistance in tumor cells [14,15,16,17,18]. Reassuringly, in earlier clinical trials assessing their use for the treatment of BPH, they were well tolerated with only reversible adverse effects in a minority of patients, including postural hypotension (4%), asthenia or light-headedness (10%), somnolence (3%) and retrograde ejaculation (8%) [19,20].

Expression and distribution of α1-adrenoreceptors has been found in the cortex, pelvis, calyces, blood vessels and tubules of the kidney, suggesting potential effects of α1 adrenoceptor antagonists in renal pathophysiology [21,22,23,24,25]. Evidence at the cellular level suggests that the antitumor effect of α1-adrenoreceptor antagonists in renal tumors proceeds via reducing vascularity and impairing growth within the tumor microenvironment (via apoptosis and overcoming anoikis resistance). In this review, we outline the mechanism of α1-adrenoceptor antagonists in targeting renal cancer epithelial and endothelial cells and the potential therapeutic efficacy of using these clinically used FDA-approved drugs for the treatment of advanced RCC.

## 2. Mechanism of Action of α-Adrenoreceptor Antagonists in Human Disease

Adrenergic receptors (adrenoreceptors) are G-Protein coupled-receptors that are distributed throughout the body. They serve as receptors for catecholamines (noradrenaline and epinephrine) secreted from the autonomic sympathetic nervous system and play an important role in the regulation of a wide range of physiological systems in the body [23,24]. Alpha (α) receptors mediate smooth muscle contraction and vasoconstriction, while beta (β) receptors mediate vasodilation, smooth muscle relaxation, bronchodilation, and excitatory cardiac function [17,21]. The α-adrenoceptors are divided into two classes: α1 and α2, both of which are present in the renal vasculature and mediate vasoconstriction of exogenous and endogenous noradrenaline [22,24]. The α1-adrenoceptors are further sub-divided into α1A, α1B, α1D, with α1A subtype of therapeutic interest because of its location in the prostate, vas deferens, and urethra in humans [21,23,24,25].

Quinazoline-derived compounds blocking α1-adrenoreceptors have been found to reduce prostatic smooth muscle tone and relieve overall obstruction, as seen by their success in treating BPH [19]. This mechanism of action is also utilized in the treatment of renal and ureteric stones, as α1 blockers reduce intra-ureteral pressure and increase fluid passage [12,26,27,28]. Remarkably in human prostatic disease, these compounds not only target the alpha1-adrenergic-receptor mediated smooth muscle contraction [29], but they can also effectively induce apoptosis of tumor epithelial and endothelial cells [17,30,31]. It is important to note that the quinazoline-derived compounds can induce apoptosis among benign prostate epithelial cells, as well as in both androgen-dependent and castration-resistant prostate cancer cells, via α1-adrenoceptor –independent mechanisms [30,31,32,33,34,35]. This supports a strong cellular basis for their pharmacologic use in other cancer types.

**Figure 1:**
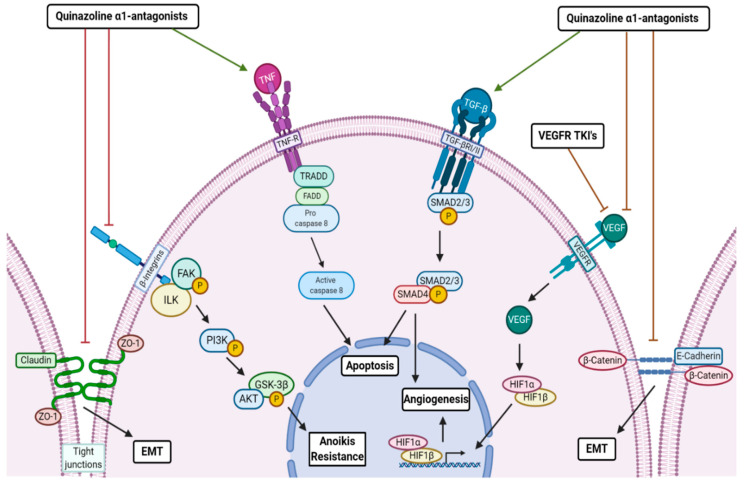
Biological Mechanisms of Anti-tumor Action of Quinazoline α1-Adrenoceptor Antagonists. Schematic diagram demonstrating the signaling mechanisms potentially targeted by quinazolne α1-andrenoceptor antagonists in attenuating renal tumor initiation and progression to metastasis. Quinazoline based α1-andrenoceptor antagonists influence the interconversion of epithelial–mesenchymal-transition (EMT) to mesenchymal–epithelial-transition (MET) phenotypes by targeting tight junctions and E-cadherin-mediates cell adherence. Tumor cells succumb to anoikis by disruption of integrin-mediated cell survival via integrin-linked kinase (ILK). Quinazoline based α1-andrenoceptor antagonists induce apoptosis by either tumor necrosis factor (TNF)-mediated Fas-associated death domain (FADD)/caspase 8 activity and DNA fragmentation and/or Smad4 activation and apoptotic gene induction by transforming growth factor (TGF)-β. Angiogenesis is inhibited by vascular endothelial growth factor receptor (VEGFR)–tyrosine kinase inhibitors and quinazoline based α1-andrenoceptor antagonists can also target tumor vascularity by disruption of VEGF-mediated HIF1 transcriptional expression and potentially TGF-β signaling.

The signaling mechanisms driving the intracellular antitumor action by quinazoline-based α1-adrenoceptor antagonists against prostate cancer epithelial and endothelial cells are summarized on Figure 1. (1) Smad activation of transforming growth factor (TGF)-β1 signaling, which controls cellular proliferation, differentiation, and apoptosis in human cancers cell including prostate cancer cells [3,30,31,32]; (2) Engaging the death receptor Fas-associated death domain (FADD)-mediated caspase-8 activation and apoptosis induction [3,32,33]; (3) Inhibition of the VEGF-mediated angiogenesis and Akt survival mechanisms navigating tumor vascularity [3,33,34]; and (4) α1-adrenoceptor antagonists have the ability to block cellular adhesion and invasion by targeting cell-cell interaction and impairing cell tight junctions (and also between epithelial and endothelial cells with the extracellular matrix; ECM), consequentially impacting epithelial–mesenchymal-transition (EMT) to mesenchymal–epithelial-transition (MET) phenotypic interconversions and increasing cellular vulnerability to anoikis (Figure 1) [3,35,36].

Moreover we previously established that integrin-linked kinase (ILK), a serine and threonine protein kinase, plays a key role in anoikis resistance by interacting with the cytoplasmic domains of β1-integrin and β3-integrin, which are pivotal in regulating cell adhesion, fibronectin–ECM assembly, and anchorage-dependent cell growth [34,35,36,37]. Within the tumor microenvironment (TME), ILK is activated in its phosphorylated form by focal adhesion kinase (FAK) and phosphatidylinositol 3-kinase (PI3-kinase)/Akt pathways [33,38,39,40]. By inhibiting ILK, quinazoline-derived α1-adrenoceptor antagonists can disrupt these cell-survival signals towards anoikis induction [3,41,42]. Considering that resistance to anoikis (and evasion of apoptosis in detached cells) is a primary contributor to cancer metastasis [43,44] and ultimately lethal disease, the ability to overcome this resistance points to a unique therapeutic value of quinazoline-derived α1-adrenoreceptor antagonists.

## 3. Antitumor Effects of α1-Adrenoceptor Antagonists

Table 1 summarizes the updated evidence from clinical, translational and epidemiological studies, suggesting the antitumor action of α1-adrenoceptor antagonists in human malignancies. The published work from our group and other investigators makes a strong case in support of the repurposing of the α1-adrenoceptor antagonists (with a good safety profile) and advance our current understanding of the clinical value of these therapeutic modalities for the treatment of GU-cancers including renal cancer

### 3.1. Prostate Cancer

Based on these pharmacological mechanisms of actions, α1-adrenoceptor antagonists have been shown to have efficacy in the treatment of several genitourinary cancers. There is mounting evidence of the effectiveness of quinazoline-derived α1 blockers in the clinical treatment patients with BPH and prostate tumors. Studies have shown that α1-adrenoceptor antagonists like prozasin and naftopidil inhibit cell growth, arrest cell cycling, decrease microvessel density, and induce apoptosis in human prostate cancer cells [34,35,45]. Doxazosin, a clinically used quinazoline-based α1-adrenoreceptor antagonist, reduced endothelial cell viability and suppressed tumor vascularity in prostate cancer xenografts. The drug additionally exhibited significant antitumor efficacy against models of metastatic castration-resistant prostate cancer (CRPC) [17,30]. In a retrospective observational cohort study at the VA Medical Center in Kentucky, Harris et al. (2007) found that in over a 5-year period in this clinical setting, exposure to quinazoline-based α1-adrenoreceptors antagonists, such as doxazosin and terazosin, significantly decreased the incidence of prostate cancer from 2.4% to 1.65%, corroborating the results of previous investigations [15,45]. While a case-control study of 23,320 men in the Finnish Cancer Registry and national prescription database found tamsulosin and alfuzosin did not improve the odds of developing prostate cancer, the study did discover the drugs significantly decreased the incidence of high-grade tumors in the cohort [47].

More recently, Hart et al. (2020) studied 303 prostate cancer patients to retrospectively determine if α1-blockers influenced response to radiotherapy for localized prostate cancer. The authors found that those treated with prazosin had a 3.9 lower relative risk of biochemical relapse. While not statistically significant, both tamsulosin and prazosin extended survival without recurrence by 13.15 and 9.21 months, respectively [48]. Furthermore, drug optimization efforts led to the development of the quinazoline-derived drug DZ-50. This novel α1 blocker has exerted chemoprotective qualities in vivo in BPH and prostate cancer cells through decreasing angiogenesis and increasing anoikis via inhibition of the TGF-β1 and insulin-like growth factor (IGF) pro-growth pathways [34,35].

### 3.2. Bladder Cancer

When evaluating antitumor activity of α1 blockers in terms of cell viability, cell cycle progression, competition, and apoptotic signaling in bladder cancer, Nakagawa et al. (2016) showed that naftopidil was one of the strongest antitumor α1-adrenoceptor antagonists [45]. Significantly enough, oral administration of naftopidil reduced tumor volume in a xenograft model in a concentration (10–100 μmol/L)-dependent manner, suggesting promising outcomes of α1 blockers in bladder cancer treatment [16]. To a lesser extent, prazosin has been shown to reduce survival of human bladder cancer cells at concentrations more than 30 μmol/L [14]. Terazosin, proven to induce apoptosis in prostate cancer cells, reduced tumor vascularity and induced apoptosis in transitional cell carcinoma (TCC) of the bladder in a retrospective case-control study using a pathological examination of specimens from patients undergoing radical cystectomy (Table 1) [49]. An independent retrospective observational study of 27,183 men confirmed these results and found that those treated with the quinazoline based adrenoceptor antagonists terazosin and doxazosin had a 43% lower relative risk of developing bladder cancer than unexposed men [50].

### 3.3. Colorectal Cancer

Epidemiological evidence from case-control studies enabled promising insights into the use of doxazosin as therapeutic and a chemopreventive strategy in treating colorectal cancer. An in vitro case-control study found that the α1 adrenoceptor antagonist, doxazosin significantly suppressed the proliferation of RKO colon cancer cell lines within human colorectal cancer cell assays. Recent pre-clinical studies demonstrated in vivo treatment of mice harboring colon cancer xenografts with doxazosin resulted in a significant decrease in tumor numbers and size compared to control untreated mice [46]. While limited, these results support the ongoing pursuit of the use of α1-adrenoreceptors antagonists in cancer treatment.

### 3.4. Adrenal Cancer

While not directly related to the genitourinary system as the other malignancies we have discussed, it is important to address the recent discoveries of the effect of α1-adrenoreceptors antagonists on adrenal cancer, specifically pheochromocytoma. While limited, there are promising preliminary results in the anti-adrenergic effects of α1-blockade in managing unchecked catecholamine production in pheochromocytoma. High circulating catecholamine levels stimulate alpha receptors on blood vessels, thereby causing vasoconstriction and increased total peripheral resistance. Thus, α-adrenergic blockade helps control blood pressure and prevent hypertensive crisis in the preoperative setting of surgical resection for metastatic pheochromocytomas [51]. While randomized controlled trials are lacking, a literature review has shown the effectiveness of doxazosin and phenoxybenzamine in the preoperative treatment of pheochromocytomas; however, further research is needed in better understanding the use of these drugs, especially in combination with β-blockers for preoperative treatment [51,52].

## 4. Potential Therapeutic Value in Renal Cancer

Original studies by our group provided initial translational insights into the therapeutic effects of α1-adrenoceptor antagonists in RCC preclinical models [3]. Doxazosin induces apoptosis in cancer cells through similar α1-adrenoreceptor-independent mechanisms as found in human prostate cancer cell models [3]. Molecular assays have demonstrated this quanizoline-based α1-adrenoceptor antagonist induces apoptosis in prostate cancer cells expressing C-Flip, an endogenous inhibitor of FADD-mediated activation, and subsequently cleaving caspase-8 [3]. As illustrated on Figure 1, doxazosin also induces apoptosis in renal cancer cells through activation of TGF-β1 signaling via Smad effector phosphorylation and targeting Akt survival mechanisms [31,32,33,34].

Additional cell-based evidence suggests that α1-blockers impair cancer progression to metastasis via anoikis induction at pharmacologically relevant doses, proceeding via an α1-adrenoreceptor-independent mechanism. Structural optimization studies led to the generation of a quinazoline-based derivative, of α1-adrenoreceptors antagonist, DZ-50, that was shown to overcome anoikis resistance in human renal cancer cells by disrupting integrin/FAK-mediated cell survival pathways in vitro and in vivo [3]. Doxazosin and DZ-50 were both found to exert potent antitumor action against human renal cancer cell lines 786-0 (harboring a VHL tumor-suppressor gene mutation and a highly angiogenic phenotype) and Caki cells (without a VHL mutation) [3].

DZ-50 has the chemoprotective potential to suppress angiogenesis and reverse the hypoxic nature of cancer through disrupting the tumor microenvironment [30]. The process of EMT, directed by TGF-β within the tumor microenvironment phenotypic landscape, confers acquisition of an invasive phenotype via resistance to anoikis, promoting angiogenesis, metastatic progression, and treatment failure. We first reported the ability of the novel quinazoline-derivative, DZ-50 to disrupt the ILK-1/integrin β1 complex and reduce phosphorylation of its downstream targets, AKT and GSK-3β [3]. As mentioned, this is an important mechanism in inducing anoikis in cancer cells because ILK regulates several integrin-mediated cellular processes, including cell adhesion, fibronectin-ECM assembly and anchorage-dependent cell growth [35,39]. By inhibiting ILK, DZ-50 is then able to kill tumor cells via blocking AKT and FAK phosphorylation and subsequent cell survival, disrupting integrin adhesion (α5β1 and α2β1), and engaging ECM associated tumor suppressors [3,30]. Through anoikis induction, DZ-50 has been found to significantly impair RCC metastasis in in vitro and in vivo models [3,17]. In vitro metastasis assays found that DZ-50 significantly decreased the adhesion potential of RCC to fibronectin and laminin in a time-dependent manner and subsequently suppressed the cells’ migratory and invasive capabilities. Mechanistic analysis of anoikis induction (determined by Annexin V-based flow cytometry) revealed that this novel agent inactivates critical cellular survival pathways through inhibition of FAK phosphorylation, inactivation of AKT and GSK-β in the focal adhesion complex signaling cascade, and disruption of integrin-mediated focal adhesion complexes, such as FAK, ILK-1 and paxillin [3]. By interfering with this survival signaling, DZ-50 successfully reverses anoikis resistance and induces cancer cell death [3,17]. By sensitizing cells to anoikis through disruption of integrin β1-mediated focal adhesion complexes, the novel quinazoline-derived agent acquires a high therapeutic value by effectively reversing anoikis resistance in metastatic RCC tumors [3]. Temporal analysis of cell death in response to DZ-50, established that anoikis occurred prior to apoptosis [3]. Furthermore, DZ-50 exerted a more potent inhibitor effect than doxazosin on ILK-1, FAK, and paxillin binding to integrin-β1 in vivo in human renal cancer 786-0 and Caki cells [3,17,18]. In both RCC cell lines, DZ-50 led to significantly greater inhibition of tumor cell adhesion, migration and invasion than doxasozin did at pharmacologically relevant doses [3]. These findings support that the structural optimization of this particular quinazoline-based α1-adrenoreceptor antagonist has furthered a promising effect in inducing anoikis and impairing renal tumor vascularity to impair metastasis. Naftopidil has also been investigated in this context, with studies demonstrating in vitro suppression of proliferation in ACHN and Caki-2 RCC cell lines [13,53]. Fluorescence-activated cell sorting (FACS) analysis revealed that renal cancer cells treated with naftopidil underwent G_1_-cell cycle arrest in vitro; the drug also decreased tumor weight and vascularity in RCC xenograft models in naftopidiol-treated excised human RCC [53]. Therefore, naftopidil provides another putative systemic therapy for the treatment and prevention of RCC that, based on this evidence, warrants further investigation.

## 5. The Repurposing of α1-Blockade in the Management of RCC

Drug repurposing refers to the development of new applications and uses for existing drugs. The advantage of this method is that the drugs under investigation have already been “de-risked,” have been approved by the FDA, have an established safety/toxicity profile and their subsequent development timelines and costs are significantly reduced. Historically, the concept of drug repurposing has been based on incidental discoveries, but a more formal approach has been proposed internationally to realize the potential in reusing currently available drugs. Emerging recommendations for integrative platforms of data analysis to systematically synthesize results from industry drug trials for more efficient discovery of new therapeutic uses and effects of novel compounds, advance combinatorial approaches for efficacy and treatment optimization. Drug repurposing has also been accelerated by the removal of patency and regulatory barriers that may prevent clinical use and the increasing funding opportunities for drug repurposing initiatives, particularly for less common diseases [54].

Quinazoline-based α1-adrenoceptor-antagonists represent an important category of drug repurposing, having already been FDA approved, with an established safety profile and extensively prescribed for the treatment of HTN and BPH for the last 30 years. Moreover, RCC patients, who have an average age at diagnosis of 65 years, commonly suffer from comorbidities including HTN and BPH (if male), notwithstanding the common association of HTN with RCC as a paraneoplastic syndrome secondary to renin and adrenocorticotropic hormone (ACTH) secretion, parenchymal or ureteral compression, and polycythaemia or an arterio-venous fistula. This would lend itself to the use of quinazoline-α1-adrenoceptor-antagonists as a logical choice in RCC patients with HTN for a potential bimodal treatment effect. Clinicians treating patients with RCC have a strong advantage to further explore such treatment and impact patient survival.

Analogous to the observational cohort study in prostate cancer discussed earlier, epidemiology studies retrospectively exploring the use of quinazoline-based α1-adrenoceptor-antagonists for the treatment of HTN or BPH for patients who were subsequently diagnosed with RCC would allow comparisons of cumulative incidence with populations of RCC patients who were unexposed, thus providing insight into the chemopreventive effects of the drug [17,46]. In a surgical setting, retrospective analyses of patient cohorts who underwent nephrectomy for renal masses with and without extensive exposure to α1-adrenoceptors for the treatment of HTN or BPH pre-operatively, as well as patients who continued α1 blockade for a period of time post-surgery, would allow an assessment of the efficacy of neoadjuvant or adjuvant quinazoline-based α1-adrenoceptor-antagonists on long-term RCC oncological outcomes. Moreover, immunohistochemical profiling of renal tumors may establish a novel anoikis signature that could correlate with the effects of α1 blockade on clinical outcome and survival in patients with high-risk RCC, potentially contributing to risk stratification and treatment decisions. Finally, applying translational research to further investigate the mechanisms of quinazoline-induced anoikis in RCC and its influence on both the tumor microenvironment and EMT may reveal additional actions on alternative signaling pathways and guide the development of combination regimes with other emerging targeted therapies.

## 6. Conclusions and Future Directions

In summary, this review accomplishes the aim of the study to investigate the effectiveness of quinazoline-based α1-adrenoceptor antagonists in the treatment of RCC by demonstrating the translational value of quinazoline-based α1-adrenoceptor-antagonists as anti-tumor-modalities with potential efficacy at all stages of the RCC patients’ journey (neoadjuvant, adjuvant, salvage and metastatic). Retrospective epidemiological studies are underway to assess the impact of quinazoline-derived α1 adrenoceptor antagonists as chemopreventive agents, and prospective clinical trials designed to investigate their efficacy in pre-surgical, post-surgical, and in-patient settings of metastatic disease. There is high translational significance in the repurposing of the α1-adrenoceptor antagonists (FDA-approved drugs) to establish their therapeutic benefit as effective treatment modalities for patients with metastatic renal cell carcinoma (RCC). Our current research efforts pursue this drug repurposing at three levels: (a) the mechanistic level, by interrogating the functional exchanges between anoikis signaling and phenotypic EMT within the kidney TME to define novel mechanisms of action; (b) the translational level by directly examing precision combination therapies in pre-clinical models of RCC with and without VHL mutations; and (c). at the clinical setting by undertaking retrospective epidemiological studies to determine the impact of the use of quinazoline-derived α1 adrenoceptor antagonists as chemopreventive agents in RCC cancan also by prospective clinical trials designed to investigate their efficacy in pre-surgical, post-surgical, and in-patient settings of RCC patients with metastatic disease [54]. If such investigative efforts demonstrate clear efficacy, RCC patients with advanced disease can therapeutically benefit from their clinical use in the near future. With the international initiatives in place encouraging the use of repurposed drugs, the introduction of new, effective RCC treatment modalities based on α1-blockade can rapidly be integrated into clinical use and markedly improve oncological outcomes of RCC patients.

## Figures and Tables

**Table 1:** Therapeutic Impact of α1-Adrenoreceptor Antagonists Use against Human Cancers.

Drug	Neoplasm	Effect	Cellular Mechanism
*Naftopidil, Prazosin* [14,45]	Bladder Cancer	Inhibit cell growth and viability in vitro in ACHN human cell lines	- Induce apoptosis via caspase activity
*Doxazosin* [46]	Colorectal Cancer	Decrease tumor numbers and size in vitro in RKO human cell lines and in vivo in mouse models	- Induce apoptosis via caspase activity
*Doxazosin, Naftopidil, Prozasin, Terozasin, DZ-50* [14,17,32,35,45]	Prostate Cancer	Reduce cell viability and tumor vascularity in vitro and in vivo, including in castration-resistant prostate cancer	- G2 checkpoint arrest Inhibit cell growth Decrease microvessel density Induce apoptosis via caspase activity, Smad activation of TGF- β1 signaling (*Doxazosin)* Induce anoikis by disrupting integrin-mediated cell survival pathways (*DZ-50)*
*Doxazosin, Naftodipil, DZ-50* [3,45]	RCC	Inhibit cell proliferation and reduce vascularity in vitro and in vivo in lines with and without VHL mutation	- Induce apoptosis by disabling FADD inhibitors, Smad activation of TGF- β1 signaling (*Dozazosin)* G1 cell cycle induction arrested in tumor and vascular epithelial cells (*Naftopidil)* Induce anoikis by disrupting integrin-mediated cell survival pathways (*DZ-50)*
*Terazosin* [46]	TCC	Reduce tumor vascularity and cell growth in vivo	- Induce apoptosis and decrease microvessel density

## Abbreviations

ACTH	Adrenocorticotropic Hormone
ASCO	American Society of Clinical Oncology
BPH	Benign Prostatic Hyperplasia
CRPC	Castration-Resistant Prostate Cancer
ECM	Extracellular Matrix
EMT	Epithelial–Mesenchymal Transition
MET	Mesennchymal–Epithelial-Transition
FACS	Fluorescence-activated cell sorting
FADD	Fas-Associated Death Domain
FAK	Focal Adhesion Kinase
FDA	Food and Drug Administration
HIF-α	Hypoxia-Inducible Factor-α
HTN	Hypertension
IGF	Insulin-Like Growth Factor
ILK	Integrin-Linked Kinase
PI3-K	Phosphatidylinositol 3-Kinase
RCC	Renal Cell Carcinoma
TCC	Transitional Cell Carcinoma (of the bladder)
TGF-β	Transforming Growth Factor-β
TKI	Tyrosine Kinase Inhibitors
TME	Tumor Microenvironment
TNF	Tumor Necrosis Factor
VHL	von Hippel-Lindau
VEGF	Vascular Endothelial Growth Factor
VEGFR	Vascular Endothelial Growth Factor Receptor
